# cGAS and STING: At the intersection of DNA and RNA virus-sensing networks

**DOI:** 10.1371/journal.ppat.1007148

**Published:** 2018-08-16

**Authors:** Guoxin Ni, Zhe Ma, Blossom Damania

**Affiliations:** 1 Lineberger Comprehensive Cancer Center, University of North Carolina at Chapel Hill, Chapel Hill, North Carolina, United States of America; 2 Department of Microbiology and Immunology, University of North Carolina at Chapel Hill, Chapel Hill, North Carolina, United States of America; Mount Sinai School of Medicine, UNITED STATES

## Innate immune sensing of RNA and DNA viruses

As the first line of host defense, the innate immune system utilizes germline-encoded receptors named pattern-recognition receptors (PRRs) to detect invading pathogens. PRRs recognize conserved molecular structures of pathogens known as pathogen-associated molecular patterns (PAMPs) to initiate immune responses that counteract pathogen infection. The immunostimulatory feature of exogenous nucleic acids, such as viral DNA and RNA, has been known for more than half a century, but the mechanism by which they function as an immune stimulant remained unclear for a long time. The past two decades have witnessed tremendous progress in understanding the signaling mechanisms of innate immune networks and established the retinoic acid inducible gene-I (RIG-I)/melanoma differentiation associated gene 5 (MDA5)–mitochondrial antiviral-signaling protein (MAVS) axis and cyclic GMP-AMP synthase (cGAS)–stimulator of interferon genes (STING) axis as the major sensing pathways for cytosolic RNA and DNA, respectively [1]. However, emerging evidence indicates that, in addition to its well-established role in sensing cytosolic DNA, the cGAS–STING pathway is also involved in restricting RNA virus infection, suggesting that there exists crosstalk between the innate sensing of cytosolic DNA and RNA.

## Canonical role of the cGAS–STING pathway in sensing DNA virus infection

**Fig 1:**
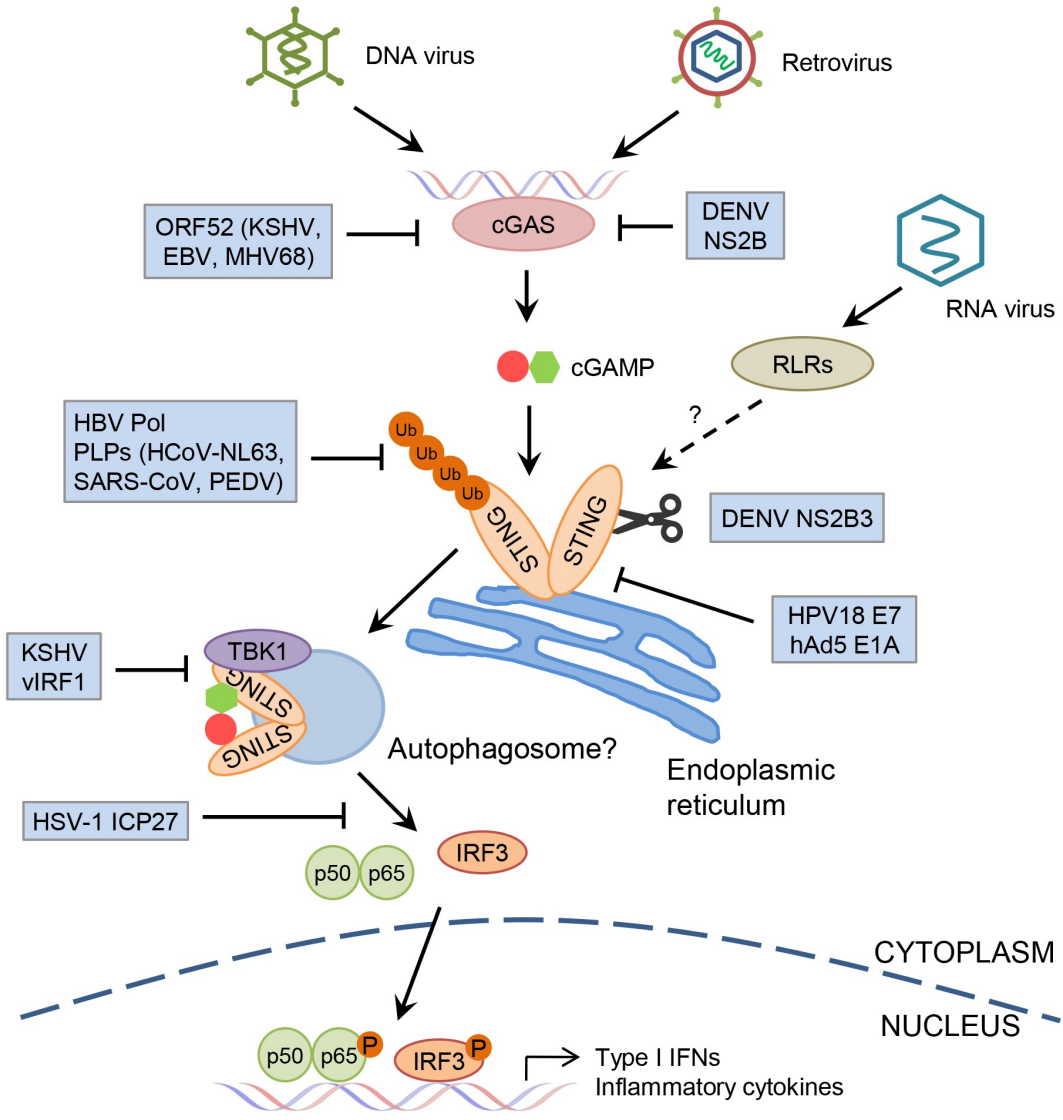
The cGAS–STING pathway and its counteraction by viruses. Genomic DNA form DNA viruses or reverse transcription intermediates from retroviruses are recognized by cGAS, which catalyzes the production of cGAMP to bind and activate the ER-resident adaptor protein STING. STING then forms a complex with TBK1 and translocates from the ER to the perinuclear lysosomal compartments via an autophagy-like process. The STING–TBK1 complex subsequently activates transcription factors IRF3 and NF-κB to induce the production of type I IFNs and inflammatory cytokines to establish an antiviral state. Viruses have developed numerous strategies to antagonize the cGAS–STING pathway. Tegument protein ORF52 from gammaherpesviruses inhibits cGAS binding to viral DNA, while nonstructural protein NS2B of DENV promotes cGAS degradation. Similarly, DENV NS2B3 protease cleaves STING and leads to its degradation. HBV polymerase and papain-like proteases of human coronaviruses prevent or remove the K63-linked Ub of STING. KSHV vIRF1 blocks the TBK1-mediated phosphorylation of STING, while HSV-1 ICP27 prevents the phosphorylation of IRF3 by TBK1. HPV18 E7 protein and hAd5 E1A protein bind to STING and inhibit its activation. cGAMP, cyclic GMP-AMP; cGAS, cyclic GMP-AMP synthase; DENV, Dengue virus; ER, endoplasmic reticulum; HBV, Hepatitis B virus; hAd5, human adenovirus 5; HSV-1, herpes simplex virus 1; HPV18, human papillomavirus 18; ICP27, infected cell protein 27; IFN, interferon; IRF3, interferon regulatory factor 3; KSHV, Kaposi's sarcoma-associated herpesvirus; NF-κB, nuclear factor-κB; NS2B, nonstructural protein 2B; ORF52, open reading frame 52; P, phosphorylation; RLRs, RIG-I-like receptors; STING, stimulator of interferon genes; TBK1, TANK binding kinase 1; Ub, ubiquitination; vIRF1, viral interferon regulatory factor 1.

cGAS binds to cytosolic double-stranded DNA (dsDNA) from various sources, including bacteria, DNA viruses, and retroviruses, in a sequence-independent but length-dependent manner [1, 2]. Following the binding of dsDNA, cGAS catalyzes the production of a second messenger known as cyclic guanosine monophosphate (GMP)-adenosine monophosphate (AMP) (cGAMP) in the presence of GTP and ATP, which subsequently binds to the adaptor protein STING on the endoplasmic reticulum (ER) membrane [1]. Aside from cGAMP, STING also directly senses other cyclic dinucleotides (CDNs), which are secreted by some bacteria [3]. After binding to CDNs, the STING dimer undergoes a dramatic trafficking process from the ER to the Golgi complex and eventually to perinuclear compartments to form large punctate structures where it is degraded [4]. STING recruits TANK binding kinase 1 (TBK1) and activates transcription factors interferon regulatory factor 3 (IRF3) and nuclear factor-κB (NF-κB), which then translocate into the nucleus to induce the transcriptional activation of type I interferons (IFNs) and other inflammatory cytokines, thus establishing an antiviral state in infected and uninfected neighboring host cells [4] (Fig 1). Numerous DNA viruses have been reported to activate the cGAS–STING pathway, and cGAS or STING deficient mice are more susceptible to lethal infection after exposure to many DNA viruses, including herpes simplex virus 1 (HSV-1), vaccinia virus, and murine gammaherpesvirus 68 (MHV68) [5]. Infection of retroviruses such as human immunodeficiency virus (HIV) generates RNA:DNA hybrids and dsDNA in the cytosol that can also activate the cGAS–STING pathway [6, 7]).

## Noncanonical role of the cGAS–STING pathway in restricting RNA virus infection

The question of whether cGAS and STING are also engaged in antiviral responses to RNA viruses has been asked since the very beginning. A quick answer to this question would be yes because studies have shown that deficiency of cGAS or STING in cells or mice greatly facilitated replication of several RNA viruses, such as vesicular stomatitis virus (VSV), Sendai virus (SeV), dengue virus (DENV), and West Nile virus (WNV) [8–11]. However, exactly how cGAS and STING are involved in RNA virus-induced immune responses is largely unknown. Although cGAS was reported to bind dsRNA, this interaction did not lead to the production of cGAMP [12]. Moreover, cGAS deficiency does not affect SeV-induced IFNβ production [13]. Therefore, although cGAS restricts replication of some RNA viruses, it is not required for RNA virus-induced type I IFN responses. One recent study reported that DENV infection led to mitochondria damage and release of mitochondrial DNA into the cytosol, which then activated the cGAS–STING pathway to potentiate host defense responses [14]. This finding provides a possibility that cGAS might play an indirect role in restricting RNA virus infection.

STING was found to interact with RIG-I and MAVS [15, 16], which are key components of the RNA sensing pathway, indicating that it might play a role in RNA virus-induced cytokine production. Some studies showed that loss of STING significantly impaired VSV and SeV-induced IFNβ production [8, 16], while others reported that cells produced type I IFNs normally after VSV or SeV infection in the absence of STING [11, 17]. Nevertheless, STING is required for the induction of some antiviral cytokines other than type I IFNs, such as C-C motif chemokine ligand 2 (CCL2) and C-C motif chemokine ligand 20 (CCL20), in response to SeV and VSV infection [17]. Recently, STING, but not cGAS, was found to be required for full interferon production induced by enveloped RNA viruses such as influenza A virus (IAV) [18]. Taken together, the role of STING in RNA virus-mediated type I IFN and cytokine production still awaits further investigation, and it might be virus or cell type specific.

DNA virus infection leads to quick ubiquitination and phosphorylation of STING, which is required for its trafficking and subsequent degradation in the perinuclear lysosomal compartment [19, 20]. However, RNA virus infection does not cause any post-translational modifications nor the degradation of STING [11, 20]. On the contrary, infection of some RNA viruses was seen to up-regulate expression of STING at both mRNA and protein levels [21]. Therefore, distinct and context-dependent mechanisms likely exist between STING-mediated antiviral responses to DNA versus RNA viruses. The concerted role between RIG-I and STING in RNA virus-induced defense responses has been reported [15, 21], but the underlying mechanism is unclear. A recent study demonstrated that rather than induce IFN expression, STING initiates a global translation inhibition to restrict production of both viral and host proteins in a RIG-I/MDA5-dependent but MAVS-independent manner [11]. Thus, recognition of RNA virus infection by RIG-I/MDA5 probably results in two distinct responses: one is mediated by MAVS to induce IFNs and cytokines, and the other is mediated by STING to inhibit translation.

## Evasion of the cGAS–STING pathway by DNA and RNA viruses

Considering the central role of cGAS and STING in the innate DNA sensing pathway, it’s not surprising to find that many DNA viruses have evolved effective strategies to antagonize the function of cGAS and STING in order to facilitate their replication in host cells [5] (Fig 1). Binding of viral DNA is the first step of the DNA sensing pathway. The tegument protein open reading frame 52 (ORF52) of several gammaherpesviruses, including Kaposi's sarcoma-associated herpesvirus (KSHV), Epstein–Barr virus (EBV), and MHV68, was found to interact with cGAS and disrupt its binding to viral DNA, thus inhibiting activation of the cGAS–STING pathway [22]. Another KSHV protein, latency-associated nuclear antigen (LANA), was also reported to interact with cGAS and antagonize the cGAS–STING-dependent signaling [23]. STING seems to be an even more favorable target of many DNA viruses, probably because of its essential role in transducing signaling from not only cGAS but also other DNA sensors [1]. HSV-1 regulatory protein infected cell protein 27 (ICP27) interacts with STING and TBK1 and thereby prevents the phosphorylation of IRF3 by TBK1 [24]. Another HSV-1 protein ICP0 was reported to promote degradation of interferon-γ-inducible protein 16 (IFI16), which detects HSV-1 DNA in human fibroblasts, thereby blocking its activation of downstream STING-dependent signaling [25]. Interestingly, HSV-1 appears to induce infected cells to export exosomes containing STING [26]. KSHV viral interferon regulatory factor 1 (vIRF1) was also found to interact with STING and prevent its interaction with TBK1, thereby inhibiting TBK1-mediated phosphorylation and activation of STING [27]. Two oncoviruses, human papillomavirus 18 (HPV18) and human adenovirus 5 (hAd5), have been shown to inhibit STING activity using their viral oncoproteins E7 and E1A, respectively [28]. Moreover, the Hepatitis B virus (HBV) polymerase was found to bind STING and attenuate its K63-linked polyubiquitination and function [29].

Emerging evidence also indicates that RNA viruses have developed their own strategies to antagonize cGAS and STING activity (Fig 1). The nonstructural protein NS4B of yellow fever virus (YFV) was the first reported viral protein that interacts and inhibits STING, although the mechanism is unclear [8]. Additional studies showed that Hepatitis C virus (HCV) NS4B, a homolog of YFV NS4B, also disrupts STING signaling by attenuating STING–TBK1 interaction [30]. Despite also encoding a NS4B protein, DENV employs another distinct strategy to antagonize STING activity. The DENV NS2B3 protease complex specifically cleaves human STING protein but not mouse STING, leading to STING degradation and attenuation of type I IFN production [31]. Interestingly, DENV NS2B was recently found to also interact with cGAS and promote its degradation via an autophagy-dependent pathway [9]. The papain-like proteases (PLPs) from several coronaviruses such as SARS–CoV, human coronavirus NL63 (HCoV-NL63), and porcine epidemic diarrhea virus (PEDV) have been shown to associate with STING and block its dimerization and K63-linked ubiquitination, thereby inhibiting the production of IFNβ [5]. Moreover, IAV hemagglutinin inhibits STING dimerization and TBK1 phosphorylation, thereby blocking STING-dependent IFN production and facilitating IAV replication [18].

## Concluding remarks and future directions

Although it is clear that cGAS is essential in cytosolic recognition of DNA viruses, its role in restricting RNA virus infection is still inconclusive. On the other hand, it is now apparent that STING is required for host responses against both DNA and RNA viruses. Studies of mechanisms employed by STING to restrict RNA virus infection have only just begun. The study which unraveled STING’s function in protein translation inhibition has shed light on our understanding of how the cGAS–STING pathway functions in RNA virus restriction [11]. However, the underlying mechanisms still need to be further elucidated. Several outstanding questions remain: How does RIG-I/MDA5 transduce a signal to STING after sensing viral RNA? Are there any other cofactors involved in this process? It is known that STING activation requires binding of CDNs. However, cytosolic RNA does not activate cGAS to generate cGAMP, and STING does not undergo any posttranslational modifications or trafficking during RNA virus infection. This begs the question as to how STING is activated to initiate translation inhibition. Furthermore, what is the strategy that STING uses to inhibit translation if it is eIF2α-independent? Answers to these questions and others will further expand our understanding of how the cGAS–STING pathway deploys immune defenses to detect and eliminate viral infection and how viruses evade or inhibit activation of this pathway. Lessons learned from this will greatly facilitate the development of new vaccines and antivirals for the prevention and treatment of infectious diseases.
